# Effects of regularly consuming dietary fibre rich soluble cocoa products on bowel habits in healthy subjects: a free-living, two-stage, randomized, crossover, single-blind intervention

**DOI:** 10.1186/1743-7075-9-33

**Published:** 2012-04-18

**Authors:** Beatriz Sarriá, Sara Martínez-López, Aránzazu Fernández-Espinosa, Miren Gómez-Juaristi, Luis Goya, Raquel Mateos, Laura Bravo

**Affiliations:** 1 Department of Metabolism and Nutrition. Institute of Food Science, Technology and Nutrition (ICTAN), Spanish National Research Council (CSIC), José Antonio Nováis 10, 28040 Madrid, Spain

**Keywords:** Dietary fibre, Cocoa, Bowel function, Gastrointestinal transit time

## Abstract

**Background:**

Dietary fibre is both preventive and therapeutic for bowel functional diseases. Soluble cocoa products are good sources of dietary fibre that may be supplemented with this dietary component. This study assessed the effects of regularly consuming two soluble cocoa products (A and B) with different non-starch polysaccharides levels (NSP, 15.1 and 22.0% w/w, respectively) on bowel habits using subjective intestinal function and symptom questionnaires, a daily diary and a faecal marker in healthy individuals.

**Methods:**

A free-living, two-stage, randomized, crossover, single-blind intervention was carried out in 44 healthy men and women, between 18-55 y old, who had not taken dietary supplements,

laxatives, or antibiotics six months before the start of the study. In the four-week-long intervention stages, separated by a three-week-wash-out stage, two servings of A and B, that provided 2.26 vs. 6.60 g/day of NSP respectively, were taken. In each stage, volunteers' diet was recorded using a 72-h food intake report.

**Results:**

Regularly consuming cocoa A and B increased fibre intake, although only cocoa B significantly increased fibre intake (p < 0.001) with respect to the non-cocoa stage. No changes in body weight were observed in either of the 4 week interventions. With cocoa product B, the number of daily bowel movements increased (p = 0.002), the frequency of having a bowel movement once a day increased (p = 0.009), the time to have a bowel movement was lower (p = 0.016) as well as the feeling of constipation (p = 0.046) without inducing adverse gastrointestinal symptoms, only flatulence increased (p = 0.019).

**Conclusions:**

Regular consumption of the cocoa products increases dietary fibre intake to recommended levels and product B improves bowel habits. The use of both objective and subjective assessments to evaluate the effects of food on bowel habits is recommended.

## Background

Dietary fibre (DF) has a broad range of beneficial health applications such as the treatment of colonic disorders, lowering risk of heart disease, diabetes and colon cancer, improving measures of glycaemic control and gastrointestinal function, etc. [1-4]. Many of these effects have been based on short-term studies being the evidence obtained from long term studies scarce [2-4]. Health authorities have already approved health claims on the benefits of DF intake and disease prevention. However, the main body of epidemiological and experimental data justifying the health benefits of DF derives from studies based on fruit, vegetables and whole grain, [5] whereas the recommended consumption of these food items is rarely reached. The average daily intake of total DF in Europe and the USA lies between 15 and 20 g, while the recommended intakes for fibre are reported to be 38 g/day and 25 g/day for men and women, respectively [6]. The new Codex definition of DF [7] acknowledges the difference between naturally occurring carbohydrate polymers, which are neither digested nor absorbed in the human small intestine, and synthetic or extracted polymers. There is some evidence of health benefits of extracted and synthetic fibres in terms of lowering levels of cardiovascular risk factors and improving measures of glycaemic control and gastrointestinal function, although the confirmation of clinical benefit is lacking [5]. In this sense, if the extracted or synthetic fibre is used as a supplement it is relevant to assess the effects of the food matrix and dose of fibre used when assessing the possible physiological benefit.

The cocoa bean has a seed coat or bran with a high content in DF, particularly insoluble fibre [8]. The composition of cocoa-derived products depends on the botanical variety as well as on genetic, agronomical and food processing. In contrast to chocolate production, the bran remains present in cocoa powder, thus being a good source of DF [9]. Moreover, this food stuff may be supplemented with fibre without negatively affecting its organoleptic properties. Cocoa powder taken as a beverage is widely consumed in many different countries, including Spain [10], being popular in different population groups, particularly as part of the breakfast meal or consumed between lunch and dinner meals.

Dietary fibre is both preventive and therapeutic for a range of common large-bowel functional diseases. Insoluble fibre polysaccharides increase stool bulk, shorten gut transit time and retain moisture in the faecal stream as a consequence of being only partially fermented by the colonic microbiota. On the other hand, soluble fibres are readily fermented but also facilitate laxation via mechanisms involving an increase in faecal biomass excretion [11,12]. It should not be disregarded that increasing the intake of DF has been associated with negative gastrointestinal effects such as flatulence, diarrhoea, bloating, etc. [13,14]. In order to evaluate the extent of the physiological effects of DF on bowel function in epidemiological studies and therapeutic trials, self-reported symptom and bowel function questionnaires have been used [1,4,15-18] in which subjective answers are usually positioned on a scale. However, bowel habits can be perceived in many different ways and thus it would be interesting to contrast the subjective data obtained with different questionnaires, as well as with an objective assessment tool such as faecal markers, radiopaque pellets, or to use certain questionnaires that formulate objective questions such as the daily diary by Bellini et al. [19]. This evaluation is also relevant in the case of functional foods rich in dietary fibre.

This study is aimed at assessing the effects of regularly consuming two soluble cocoa products with different contents of NSP on dietary fibre intake and gastrointestinal function using subjective questionnaires, an objective faecal marker and a daily diary, and to contrast the results obtained with the different assessment tools.

## Methods

### Subjects

This study was conducted according to the guidelines laid down in the Declaration of Helsinki and all procedures involving human subjects were approved by the Clinical Research Ethics Committee of Hospital Universitario Puerta de Hierro Majadahonda in Madrid (Spain). Volunteer recruitment was carried out through placing advertisements in the Universidad Complutense campus as well as through giving short talks between lectures. The inclusion criteria was: Non-vegetarians, non-smoker, non-pregnant, healthy men and women, between 18 and 55 y old, not suffering from any chronic pathology or gastrointestinal disorders or bowel abnormalities such as: irritable bowel syndrome, Crohn's diseases, ulcerative colitis, chronic intestinal inflammation, chronic constipation, diarrhoea, haemorrhoids, not suffering lactose intolerance or other food allergies. None had taken dietary supplements, laxatives, any type of medication that could affect gut motility or antibiotics six months before the start of the study. Their body mass index was not higher than 30 kg/m^2^. Fifty subjects initially accepted to participate in the study and gave informed written consent.

This work forms part of a larger study in which the effects of regularly consuming the cocoa products on cardiovascular health is assessed. The variable used to calculate the power of the study was total cholesterol. A sample size of 38 patients to take part in this crossover intervention was calculated in order to obtain a probability of 80% that the study will detect a treatment difference at a two sided 0.05 significance level, if the true difference between the treatments were 10.0 units, assuming that the within-subject standard deviation of the response variable is 15. Studies similar to the present work have been performed in smaller groups, 25 and 23 subjects, in Jenkins et al. [9] and Vuksan et al. [1], respectively, or similar size populations [17]. Fifty subjects were recruited in order to increase the power of the study and account for possible abandoners.

### Study design

The study is a free-living, two-stage, randomized, crossover, single-blind intervention. It consisted in a one-week run-in stage and two four-week long intervention stages (Cocoa product A and Cocoa product B) separated by a three-week wash-out period (Non-cocoa). The trial was held during the autumn months. Participants were randomly assigned to start taking cocoa product A or B following simple randomization procedures (computerized random numbers).

Two soluble cocoa products provided by Nutrexpa S.L. were used: A and B containing two different levels of total NSP: 15.09 and 22.00% (w/w), respectively. Cocoa product B was supplemented with extracted cocoa bran. Each serving was individually packed in identical sealed sachets that were labelled A or B. The two servings of A and B weighed 15 g and 30 g, respectively, and provided 2.26 vs. 6.60 g/day of NSP, respectively. The servings used in this study correspond to the quantity of the product that can reasonably be expected to be consumed in a day. Volunteers were indicated to consume the two servings of either product daily in 200 mL of semi-skimmed milk, thus following realistic consumption rates. The first cocoa beverage was taken at breakfast and the second between lunch and dinner, in order to reduce interferences with other food items and reproduce real consumption patterns. During the study, certain vegetables and fruits (oranges, tangerines, apples, grapes, strawberries and any other type of berry, beets and onions) were restricted in order to reduce interindividual differences in DF intake. Cocoa products A and B contained different levels of total fat: 6.2 and 3.3% (w/w), respectively (data provided by the manufacturer), however the amount provided with each serving was similar (0.93 g and 0.99 g for product A and B, respectively).

### Dietary control and compliance

Subjects were asked to maintain the same dietary habits along the study periods. Subject's dietary intake was regularly evaluated to record any possible changes in dietary habits. Volunteers were instructed on how to fill in the dietary records before starting the study and during the first intervention stage. In the last week of each study period, volunteers were asked to complete a 72 hour detailed food intake report, specifying the ingredients and amounts of food consumed and serving weights. Compliance was evaluated through counting the number of cocoa servings provided to the volunteers before and after each intervention stage, as well as through weekly calling the volunteers. In order to assess dietary composition the programme Dial (Department of Nutrition and Bromatology I, Phaculty of Pharmacy, Universidad Complutense de Madrid) was used.

### Bowel function questionnaire, symptom diary, daily diary questionnaires, faecal marker

The influence of consuming the soluble cocoa on gastrointestinal habits was assessed using two subjective questionnaires, in order to gather the maximum information on the effect of consuming the cocoa products on bowel function and to cross check the reproducibility of the answers obtained. Moreover, the answers were contrasted with a daily diary and an objective assessment tool. With this purpose, adapted versions of the subjective bowel function questionnaire by Griffenberg et al. [18] and the symptom diary by Jenkins et al. [9] were used during the last week of each stage of the study. Also the daily diary by Bellini et al. [19] which formulates objective and subjective questions was applied. Griffenberg et al. [18] questionnaire was developed to determine patients' perceptions of their bowel habits prior and post surgery (statements regarding surgery have been withdrawn in the present study), having its content been validated. It consists of a list of statements describing personal bowel habits that contain a blank that must be filled in with words: never, rarely, sometimes, frequently and always. For statistical treatment of the results, the five appreciations have been converted into a 0-4-point scale where 0 = never, 1 = rarely, 2 = sometimes, 3 = frequently and 4 = always. Similarly, with the symptom diary a 5-point scale was used to answer the questions, where 0 indicates no gas, no bloating, watery, easy to pass and no pain, and 5 indicates, severe, hard or difficult, where appropriate. In contrast, in the daily diary questionnaire, patients were asked to record the number of bowel movements per day and give yes/no responses. For statistical purposes, the conversion of yes = 1 and no = 0 was applied, and for all questions the mean was calculated per week.

For a more objective assessment of the effect of cocoa consumption on intestinal transit time, on the first day of the last week of each stage of the study the faecal marker Brilliant Blue FCF (Warner Jenkinson Europe, UK) [20] was administered in a capsule (50 mg) together with a standardised breakfast consisting of two slices of white bread and a glass of half-skimmed milk with (intervention periods) or without cocoa (non-cocoa stage). Volunteers were asked to record the appearance and disappearance time of the marker in faeces since the time the breakfast was consumed.

### Physical activity and body weight

Participants were asked to maintain their usual level of physical activity during the study. Volunteers filled in a questionnaire before starting the study to know their occupation and consequently the physical activity involved. At the beginning and the end of the study physical activity was calculated using an adapted version of the Minnesota Leisure Time Physical Activity Questionnarie by Martínez-González et al. [21]. Total energy expenditure from leisure time was obtained making the assumption that 1 MET (metabolic equivalent) is approximately 1 kcal/min for a 70-kg man. Taking this conversion factor and knowing the number of minutes that the activities were performed per day, the data was expressed as kcal/day. Volunteer's body weight was measured at the beginning and the end of the two intervention stages (Cocoa product A and Cocoa product B).

### Cocoa fibre analysis

The NSP content of the two cocoa products was analysed in triplicate defatted samples following the method of Saura-Calixto et al [22] modified in our laboratory. Briefly, to 300 mg of cocoa powder was added 0.2 mL of a pepsin solution (Cat. No. 7190, Merck, Darmstadt, Germany) containing 300 mg of pepsin/mL of HCl-KCl (0.05 M HCl and 0.03 M KCl, respectively) buffer, pH 1.5. Samples were incubated for 1 h at 40°C in a water bath with constant shaking. Then, 9 mL of Tris-maleate buffer (0.1 M, pH 6.9) was added and the pH checked. α-Amylase (1 mL of a 120 mg/mL solution in Tris-maleate buffer; Sigma, A-3176) was added, and the samples were incubated in a water bath at 37°C for 16 h with constant shaking. Samples were centrifuged (15 min, 3000 g) and supernatants removed. Residues were washed twice with 10 mL of distilled water and all supernatants combined. These were transferred into dialysis tubes (12000-14000 MWCO, Dialysis Tubing Visking, Medicell International Ltd., London, UK), and dialysed against water for 48 h at 25°C (water flow 7 L/h in a 43 L reservoir). Dialysates (soluble dietary fibre, SDF) were hydrolysed with 1 M sulphuric acid at 100°C, 90 min and NSP determined in the hydrolysate. The residues obtained after enzymatic hydrolysis of samples (insoluble dietary fibre, IDF) were washed with acetone (Panreac, 361007) and dried overnight at room temperature, and hydrolysed with sulphuric acid (12 M H_2_SO_4_, 1 h, 30°C, then diluted to 1 M H_2_SO_4 _100°C, 90 min with shaking). After acid hydrolysis, samples were centrifuged (15 min, 3000 g), pellets washed twice with distilled water, and combined supernatants collected for NSP determination.

*Uronic acids *(UA) in hydrolysates from both SDF and IDF were quantified spectrophotometrically [23] using galacturonic acid as standard. Neutral sugars (NS) were analysed by spectrophotometry following the method Southgate [24]. NSP was calculated as soluble NSP + insoluble NSP; Soluble NSP was calculated as UA + NS, and insoluble NSP as UA + NS.

### Statistical analysis

Before hypothesis testing, data were examined for normality and for non-normally distribution. The total NSP intake was initially used as a covariate considering that it was significantly different (p < 0.001) in the three stages of the study and the important role that this dietary component plays on bowel function, however as NSP effects was not significant in the majority of the parameters studied, NSP was not used as a covariate in the statistical analysis performed. In order to determine the effects of consuming the soluble cocoa products on dietary intake, on daily bowel movements and on intestinal transit time, a mixed model with repeated measures (stage) with one factorial fixed factor (cocoa product) was applied. Results from the symptom diary and bowel function questionnaire were studied using generalized estimating equations with repeated measures (stage) and one fixed factor (cocoa product). In all parameters, where there was a significant main effect, *post hoc *Bonferroni tests were also used to analyse differences between means. Statistical significance was set at p < 0.05. Statistical analysis was undertaken with SPSS statistical package (version 18.0). Data are presented as means ± standard error of mean, unless specified otherwise.

## Results

### Subjects

Of the fifty volunteers who enrolled in the study, for personal, health or professional reasons 6 subjects withdrew from the study. Of the 44 volunteers remaining, 23 were women with an average age of 25.75 y (SD 6.29) and body mass index (BMI) of 22.2 (SD 2.42) kg/m^2 ^and 21 were men with an average age of 31.50 y (SD 10.04) and BMI of 25.04 (SD 3.94) kg/m^2^.

### Cocoa product fibre analysis

The NSP and polyphenol composition of the two soluble cocoa products is shown in Table 1 (n = 3). As it can be seen, cocoa product B had a higher NSP content in comparison with product A. Although both cocoas were made of mainly insoluble NSP, cocoa A was comparatively richer than product B in soluble NSP with up to 20.74% of the total NSP as soluble NS plus UA in comparison with only 7.64% of soluble NSP in cocoa product B. On the other hand cocoa product A was richer in total polyphenols as compared to cocoa B.

**Table 1:** Non-starch polysaccharide (NSP) and polyphenol composition of the two soluble cocoa products (Mean values (n = 3) and standard deviation (SD)

	Cocoa product A (g/100 g)		Cocoa product B (g/100 g)	
	
	Mean	SD	Mean	SD
NSP composition*				
Soluble NSP	3.13		1.68	
Neutral sugars	2.46	0.43	0.69	0.04
Uronic acid	0.67	0.16	0.99	0.09
Insoluble NSP	11.96		20.32	
Neutral sugars	10.49	0.96	19.06	1.60
Uronic acid	1.47	0.09	1.26	0.07
Total NSP	15.09		22.00	

Polyphenol composition* (μg equivalent gallic acid/g)	34.04	2.28	15.75	0.67

* dry matter

### Dietary recording and compliance

Dietary compliance during the intervention was high according to volunteers' reports and to the number of servings that the volunteers returned having finished the intervention periods. Attending to the 72 hour detailed food intake reports and to the dietary composition assessment carried out with the programme Dial, the intake of both cocoa products statistically increased energy intake (p = 0.045) compared to when cocoa was not consumed (Table 2). Accordingly, protein intake was significantly lower (p < 0.001) in the non-cocoa stage compared to cocoa product A and B stages, as well as carbohydrate intake (p = 0.003), which was significantly lower in the non-cocoa stage versus cocoa product A and B stages (Table 2). The intake of DF showed significant differences along the study (p < 0.001; Table 3). Attending to the Bonferroni test, DF intake in cocoa product B stage was statistically higher compared to that in the non-cocoa stage (p < 0.001) and cocoa product A stage (p = 0.009). In contrast, polyphenol intake was higher in cocoa product A and B stages, compared to the non-cocoa stage, although these differences did not reach the level of statistical significance. Other dietary component intakes, such as total lipids, mono, poly and saturated fatty acids, minerals, vitamins A, C, E, D or total carotenes, did not show significant changes between the different stages of the study (data not shown).

**Table 2:** Effects of consuming the soluble cocoa products on energy, protein, carbohydrate and lipid intake (Mean values and standard error of mean (SEM), n = 44)

	Energy Intake (Kcal/day)		Protein Intake (g/day)		Carbohydrate Intake (g/day)		Lipid Intake (g/day)	
Stage	Mean	SEM	Mean	SEM	Mean	SEM	Mean	SEM
Non-cocoa	2,281.99	59.12	94.30^a^	2.51	210.97	6.78	105.73	3.23
Cocoa product A	2,465.61	74.69	110.48^bc^	3.47	239.41	7.88	106.17	4.49
Cocoa product B	2,558.57	97.91	117.64^c^	4.29	245.04	8.61	110.89	5.56

	p = 0.045		p < 0.001		p = 0.003		N.S.	

Effects were studied using a mixed model with repeated measures (stage) with one factorial fixed factor (cocoa product). Where there was a significant main effect, *post hoc *Bonferroni tests were also used to analyse differences between means.

^a,b ^Mean values within a column with unlike superscript were significantly different according to Bonferroni test.

**Table 3:** Effects of consuming the soluble cocoa products on total fibre and polyphenol intake (Mean values and standard error of mean (SEM), n = 44)

	Total Fibre Intake (g/day)		Polyphenol Intake (mg/day)	
Stage	Mean	SEM	Mean	SEM
Non-cocoa	17.29^a^	0.71	1424.71	106.92
Cocoa product A	19.86^a^	0.88	1549.90	163.32
Cocoa product B	23.91^b^	0.88	1696.43	132.23

	p < 0.001		N.S.	

Effects were studied using a mixed model with repeated measures (stage) with one factorial fixed factor (cocoa product). Where there was a significant main effect, *post hoc *Bonferroni tests were also used to analyse differences between means.

^a,b^Mean values within a column with unlike superscript were significantly different according to Bonferroni test.

### Bowel function questionnaire, symptom diary, daily diary questionnaires, faecal marker

Attending to both the subjective bowel function (Tables 4, 5 and 6) and the symptom questionnaire (Table 7), when product B was consumed, stool consistency (Table 7) and the output of hard stools (Table 4) was described to be slightly lower and accordingly soft stools slightly higher (Table 4), although none of the differences obtained with these three parameters were statistically significant. In contrast, attending to the daily diary (Table 8), the perception of lumpy or hard stools was significantly higher (p = 0.042) during the cocoa product A stage.

**Table 4:** Effects of consuming the soluble cocoa products were studied using subjective bowel function questionnaires (Mean values and standard error of mean (SEM), n = 44)

	Soft stools		Hard stools		Strain to have BM		Pain to have BM		Blood seen with BM	
Stage	Mean	SEM	Mean	SEM	Mean	SEM	Mean	SEM	Mean	SEM
Non-cocoa	1.29	0.09	2.01	0.10	1.44	0.09	0.72	0.08	0.09	0.03
Cocoa product A	1.27	0.12	2.09	0.16	1.48	0.14	0.66	0.11	0.14	0.05
Cocoa product B	1.52	0.12	1.91	0.15	1.41	0.13	0.72	0.11	0.09	0.04

	N.S.		N.S.		N.S.		N.S.		N.S.	

BM, bowel movement. A 4-point scale was used to answer where 0 = never, 1 = rarely, 2 = sometimes, 3 = frequently and 4 = always.

Effects were evaluated using a generalized estimating equations with repeated measures (stage) and one fixed factor (cocoa product) were applied. Where there was a significant main effect, *post hoc *Bonferroni tests were also used to analyse differences between means.

**Table 5:** Effects of consuming the soluble cocoa products were studied using a subjective bowel function questionnaire (Mean values and standard error of mean (SEM), n = 44)

	Feel bowel completely empty		Have BM once per day		Pass gas during day		Have crampy abdominal pain		Have haemorrhoid problems	
Stage	Mean	SEM	Mean	SEM	Mean	SEM	Mean	SEM	Mean	SEM
Non-cocoa	2.67	0.10	2.93^a^	0.10	2.14	0.09	1.20	0.08	0.39	0.06
Cocoa product A	2.73	0.11	3.09^a^	0.13	2.07	0.15	1.09	0.11	0.32	0.08
Cocoa product B	2.75	0.12	3.27^a^	0.11	2.32	0.14	1.27	0.12	0.34	0.09

	N.S.		p = 0.009		N.S.		N.S.		N.S.	

BM, bowel movement. A 4-point scale was used to answer where 0 = never, 1 = rarely, 2 = sometimes, 3 = frequently and 4 = always.

Effects were evaluated using a generalized estimating equations with repeated measures (stage) and one fixed factor (cocoa product) were applied. Where there was a significant main effect, *post hoc *Bonferroni tests were also used to analyse differences between means.

**Table 6:** Effects of consuming the soluble cocoa products were studied using a subjective bowel function questionnaire (Mean values and standard error of mean (SEM), n = 44)

	Have diarrhoea		Urgency to go to toilet for BM		Consider myself constipated		Time to have BM* (min)	
Stage	Mean	SEM	Mean	SEM	Mean	SEM	Mean	SEM
Non-cocoa	1.00^a^	0.07	3.28	0.08	1.34^a^	0.10	0.99^a^	0.10
Cocoa product A	0.79^a^	0.10	3.27	0.11	1.36^a^	0.14	0.98^a^	0.11
Cocoa product B	0.77^a^	0.09	3.43	0.09	1.16^a^	0.13	0.79^a^	0.13

	p = 0.043		N.S.		p = 0.046		p = 0.016	

BM, bowel movement. A 4-point scale was used to answer where 0 = never, 1 = rarely, 2 = sometimes, 3 = frequently and 4 = always.

Effects were evaluated using generalized estimating equations with repeated measures (stage) and one fixed factor (cocoa product) were applied. Where there was a significant main effect, *post hoc *Bonferroni tests were also used to analyse differences between means. * Time to have BM = duration of defecation.

**Table 7:** Effects of consuming the soluble cocoa products were assessed using a symptom related to bowel habits questionnaire (Mean values and standard error of mean (SEM), n = 44)

	Flatulence		Bloating		Stool consistency		Ease of movement		Abdominal Pain	
Stage	Mean	SEM	Mean	SEM	Mean	SEM	Mean	SEM	Mean	SEM
Non-cocoa	2.14^a^	0.09	1.08	0.11	2.36	0.07	1.15	0.11	0.51	0.08
Cocoa product A	2.07^a^	0.14	1.00	0.17	2.36	0.13	1.11	0.16	0.46	0.13
Cocoa product B	2.32^a^	0.14	1.11	0.15	2.18	0.10	1.16	0.16	0.52	0.10

	p = 0.019		N.S.		N.S.		N.S.		N.S.	

A 5-point scale was used to answer where 0 indicates no gas, no bloating, watery stool consistency, easy to pass and no pain, respectively; 5 indicates, severe, hard or difficult where appropriate. Effects were evaluated using generalized estimating equations with repeated measures (stage) and one fixed factor (cocoa product) were applied. Where there was a significant main effect, post hoc Bonferroni tests were used to analyse differences between means.

**Table 8:** Effects of consuming the soluble cocoa products on daily bowel movements was assessed by a daily diary (mean week values and standard error of mean (SEM), n = 44)

	Daily Bowel movements		Straining at defecation		Feeling of incomplete and/or difficult defecation		Lumpy or hard stools	
	
Stage	Mean	SEM	Mean	SEM	Mean	SEM	Mean	SEM
Non-cocoa	1.14^a^	0.04	0.14	0.02	0.15	0.02	0.28	0.03
Cocoa product A	1.29^b^	0.04	0.18	0.02	0.19	0.02	0.32	0.03
Cocoa product B	1.30^b^	0.04	0.17	0.02	0.15	0.02	0.27	0.03

	p = 0.002		N.S.		N.S.		p = 0.042	

Volunteers were asked to record the number of bowel movements per day and give yes/no responses to the rest of the questions. For statistical purposes, the conversion of yes = 1 and no = 0 was applied. For all questions the mean was calculated per week.

Effects on daily bowel movements was studied using a mixed model with repeated measures (stage) with one factorial fixed factor (cocoa product). Effects on straining at defecation, feeling of incomplete and/or difficult defecation and on producing lumpy or hard stools were evaluated using generalized estimating equations with repeated measures (stage) and one fixed factor (cocoa product) were applied. Where there was a significant main effect, post hoc Bonferroni tests were used to analyse differences between means.

^a, b^Mean values within a column with unlike superscript values were significantly different according to Bonferroni test.

Additionally, the time to have a bowel movement (i.e. the duration of defecation) was significantly lower (p = 0.016) when product B was consumed (Table 6) which is supported with the shorter (although not statistically different) dye appearance and disappearance times (Table 9), and the slightly higher urgency (Table 6) to go to the toilet for a bowel movement observed with the cocoa product richer in fibre. Accordingly, volunteers described having a bowel movement once a day more frequently (p = 0.009; Table 5) when the cocoa product richer in NSP was consumed, considering themselves less constipated (Table 6, p = 0.046). With both products A and B slightly less haemorrhoid problems were reported (Table 5). It is noteworthy that the feeling of having diarrhoea (Table 6) was significantly smaller (p = 0.043) in both cocoa product stages compared to the non-cocoa stage.

**Table 9:** Effects of consuming the soluble cocoa products on intestinal transit time (mean values and standard error of mean (SEM), n = 44)

	Dye appearance time (hours)		Dye disappearance time (hours)	
	
Stage	Mean	SEM	Mean	SEM
Non-cocoa	31.46	1.48	71.72	2.84
Cocoa product A	35.04	2.81	76.93	6.06
Cocoa product B	30.05	1.97	67.32	4.10

	N.S.		N.S.	

Dye appearance and disappeareance time were studied using a mixed model with repeated measures (stage) with one factorial fixed factor (cocoa product).

Daily diary answers (Table 8) showed parallel results compared to the bowel function and symptom questionnaires. With either cocoa product the number of daily bowel movements significantly increased (p = 0.002) compared to the non-cocoa stage and the straining at defecation as well as the feeling of incomplete defecation was similar in the three stages.

In summary, the significant gastrointestinal effects associated to consuming cocoa product B were positive, being the only significant negative effect having greater feeling of flatulence (p = 0.019, Table 7). However this negative perception was not supported by the answers relative to having crampy abdominal pain, passing gas during the day or bloating, as none of these parameters showed significant differences.

### Physical activity and body weight

Physical activity (Kcal/day expressed as mean ± standard error of mean) tended to be lower in cocoa product A and B stages (677.97 (SEM 68.36) and 673.62 (SEM 67.62), respectively) compared to the non-cocoa stage (818.60 (SEM 58.61), not showing statistical differences. Volunteer's body weight did not change along the experiment with either cocoa product. Body weights (kg) at the beginning and the end of product A intervention were 67.7 (SD 13.20) and 68.0 (SD 13.22) and of product B intervention were 68.0 (SD 13.28) and 68.3 (SD 13.27), respectively.

## Discussion

This is a free-living study that showed that the regular addition of two servings of soluble cocoa products, containing 15.1 and 22.0% (w/w) of NSP, respectively, to a Spanish diet is an efficacious alternative to increase fibre intake to recommended levels and the product richer in NSP (22.0%, w/w) also promotes healthier bowel habits. The servings used in this study correspond to the quantity of the product that can reasonably be expected to be consumed. The consumption rate of two cocoa beverages per day reproduces real conditions in the Spanish population and may be considered moderate [[10], http://www.cacaoychocolate.com/consumoen.html]. The only dietary modification introduced in this study, apart from consuming the cocoa products, was the restriction of certain fruits and vegetables. However taking into account the restrictions, the intake of DF remained within the range estimated in the Spanish population (16.3 - 18.4 g/day) [25] and was similar to that in European countries (18.5 g/day) [26]. Up to date, there is no gold standard for fibre intake assessment being dietary food records a well accepted alternative, particularly if the influence of smoking, alcohol intake, gender and education is considered [27]. With this regard, volunteers who participated in this study were non-smokers, consumed very low amounts of alcohol and had a similar, medium-high, education level. In the non-cocoa stage, the intake of fibre (Table 2) was near to the lower limit of the various daily fibre recommendations, which range from 15 to 35 g [28]. The regular consumption of either cocoa product improved the intake of DF with respect to the non-cocoa stage, particularly that corresponding to cocoa product B. The higher intake of fibre associated to consuming cocoa product B compared to A is due both to their unlike NSP content (22.00% vs. 15.09%, respectively) and to the different net quantity consumed daily of each cocoa product (30 g of cocoa product B vs. 15 g of A) which provided 6.6 and 2.26 g/d of total NSP.

According to the bowel function and symptom questionnaires, both cocoa diets were well tolerated and volunteers did not describe adverse gastrointestinal symptoms such as abdominal pain or bloating compared to the non-cocoa period. The only negative effect described was a higher perception of flatulence when the product richer in NSP was consumed. This outcome was not completely supported with the perception of passing gas during the day, which was slightly higher in the cocoa product B stage without reaching a statistical difference compared to other stages. This symptom has been widely described as a side-effect related to fibre intake [13,14]. Excepting this result, there was a predominant positive gastrointestinal tolerance to the fibre-rich cocoa periods which may be attributed to the adaptation hypothesis, according to which a continuous intake of DF relieves negative gastrointestinal side-effects based on the equilibrium that is reached between the gas-producing and gas utilizing bacteria [29]. Other interesting outcomes associated with the consumption of product B, was the lower perception of producing less hard stools with lower stool consistency without leading to the feeling of having diarrhoea. In agreement, Castillejo et al. [17] described that a significantly higher number of parents reported a subjective improvement in stool consistency in their chronically constipated children who consumed a supplement of cocoa husk.

Four positive significant changes on bowel function over time were described associated to consuming the cocoa product richer in fibre (6.6 g/d of total NSP): a reduced perception of being constipated, a higher record of having a bowel movement per day, supported by an objective higher number of daily bowel movements, and a lower time to have the bowel movement. The higher frequency of daily bowel movements is in agreement with other studies. Jenkins et al. [9] obtained a significantly higher frequency of movements per day when a diet containing a cocoa-bran cereal (total DF 25 g/day) was consumed for 2 weeks versus a low-fibre cereal (total dietary fibre 5.6 g/day). Similarly, Dahl et al. [16] described that the addition of a moderate amount of finely processed pea hull fibre to usual food (doses ranging 20-26 g/day) helped to normalize and improve bowel function in elderly subjects resulting in a significant increase in bowel movements among other improvements of clinical symptoms of constipation. Similar effects where observed with fibre supplementation (the doses used were "age in years plus 5 g/day") in chronically constipated children [30].

In addition to changes in bowel function based on the subject's own reports recorded in the subjective questionnaires and the diary, an objective measurement of intestinal transit time was carried out which evaluates overall colonic function. The reported average transit time usually takes 5 days [31]. On one hand, we were aware that using a faecal dye to estimate intestinal transit time presents the limitation that transit time is influenced by the level of either food or fibre in the diet [32]. In order to minimize the possible confounding effect that could be produced by other food items consumed simultaneously with the dye, the dye capsule was administered with a standardised fibre-free breakfast, formed by two slices of white bread and semi-skimmed milk which was administered in each of the stages of the study. Additionally, the dye was consumed on one of the days in which the 72-h food record was filled out, so volunteers' food intake was recorded. In this sense, no differences in DF consumption were observed between the three days in each stage (data not shown). On the other hand, the advantages of using brilliant blue are that it is non-invasive, compared to other dyes its differentiation is quite unequivocal, and it does not require stool collection [33]. Our results are in agreement with the appearance rate of 24 to 36 hours and the disappearance rate between 48 and 72 hours previously described in healthy individuals [21]. The dye appearance time was about 1.5 hours shorter when cocoa product B was consumed compared to the non-cocoa stage, while the disappearance time was 4 hours shorter. However these differences did not reach the level of statistical significance. These effects may be attributed to the higher content of water insoluble forms of NSP in cocoa product B (B: 20.3% versus A: 12.0%) which are known to promote colonic mucosal health through inducing changes such as increasing faecal bulking or reducing transit time [12,34]. Therefore, the positive self-perceived symptoms previously described associated to consuming cocoa product B are supported with objective shorter intestinal transit times, although not statistically lower.

Surprisingly, product A showed higher dye appearance and disappearance times compared to the non-cocoa stage. Regarding these results, it should be noted that very high intraindividual variance was obtained in the cocoa product A stage, with abnormally high transit times in some cases, which is in agreement with the elevated variation in intestinal transit time described in other studies [17]. These results may be attributed to other life-style factors that were not possible to control in this free-living study. Walking, running, and strength training have been described to reduce gastrointestinal transit time, [35-37] although the effectiveness of physical activity in the management of constipation remains controversial. Recently, a study performed in normally active subjects, between 24-77 y, failed to prove this relationship [38]. In contrast, in a group of subjects over 40 years of age, dietary factors have little influence on defecation habits because of the stability of large bowel function, and exercise, including walking, suggested to be more useful in preventing constipation [39]. In this study, the physical activity values are obtained based on the assumption that 1 MET is approximately 1 kcal/min may have led to an overestimation of energy expenditure although this fact is not particularly relevant as the same factor was used along the study and physical activity was not different.

As cocoa products are relatively high energy food, in fact the energy intake was significantly lower (p = 0.045) at the non-cocoa stage, their addition to a diet could induce weight gain. However, the consumption of the cocoa products did not lead to an increase in body weight, without disregarding the intervention stages were 4 weeks long. These results are in agreement with previous studies in humans [40], in which the energy intake was higher in dark chocolate consumers than in nonconsumers, although BMI in the former group was lower. The association between dark chocolate consumption and lower BMI has been attributed to the physiologic activity of polyphenols, which have also been described with tea polyphenols in humans [41]. The mechanisms involved in the antiobesity effects of cocoa have been studied in rats and it seems that cocoa modulates lipid metabolism, especially by decreasing fatty acid synthesis and transport systems, and enhances part of the thermogenesis mechanism in liver and white adipose tissue [42].

The present study presents certain methodological limitations: the sample size was calculated according to total cholesterol, therefore it is not possible to know if the study was adequately powered, especially after having observed that bowel habits show high variability. Other components of the cocoa products and diet may have influenced the outcome on bowel habits. Faecal dyes cannot be quantitated in faeces.

Strengths of the study are that it is the only study that has used multiple assessments to investigate the effect of cocoa bran on bowel habits in healthy adults. To our knowledge, there are only two other human studies that focus on the same issue, one having been carried out in constipated children and thus the questionnaires were answered by their parents [17], and the other uses only one tool, the symptom diary, to evaluate the effects of cocoa bran on bowel habits [9]. Moreover, it has been carried out in 44 subjects which is a relatively high number compared to similar studies [1,9].

## Conclusion

The regular addition of two servings of cocoa products, providing 2.26 and 6.60 g of total NSP per day, to a typical Spanish diet is an efficacious alternative to increase fibre intake to recommended levels without leading to an increase in body weight when consumed during 4 weeks. Moreover, the cocoa product enriched with fibre at 22.0% reduces the time to have a bowel movement and increases the number of daily bowel movements, without inducing adverse gastrointestinal symptoms such as abdominal pain or bloating or leading to having diarrhoea, only flatulence increases. In order to obtain more accurate information on the effects of food on bowel habits, it is recommended to use both subjective self-reported questionnaires as well as objective measurements.

## Competing interests

This study was funded by Nutrexpa S. L. It may be possible that in the future this company may gain financially from the publication of this manuscript. However, nowadays there are non-competing interests, as the interpretation of data or presentation of information has not been influenced by any personal or financial relationship with the organization, which has not financed the publication of this manuscript.

## Authors' contributions

The author's responsibilities were as follows: LB conception, design of the study and data interpretation. BS contribution to the experimental design, data interpretation and writing of the manuscript. BS and SM trial coordination, statistical analysis. AF-E analysis of questionnaires. MG-J dietary fibre analysis. LG and RM contribution to the experimental design and data interpretation. All authors reviewed and agreed on the final version of the manuscript.
